# Reviews and Book Notices

**Published:** 1879-03

**Authors:** 


### Reviews and Book Notices.

ARTICLE XVII. — TRANSACTIONS OF STATE MEDICAL SOCIETIES.

1. *Transactions of the Thirty-third Annual Meeting of the Ohio State Medical Society, held at Columbus, May 14th, 15th and 16th*, 1878.

2. *Transactions of the Medical Society of the State of Pennsylvania, at its Twenty-eighth Annual Session, held at Pittsburgh, May*, 1878.

1. The transactions of the *Ohio State Medical Society* contain so much of interest that the society may justly feel proud of its achievements as reflected from the pages of this neat little volume.

The retiring president, W. H. Philips, undertook to show the shortcomings of the present practice in court, of taking the testimony of medical experts. "The parties are allowed to summon whom they please, to testify in their favor. Neither party summons a witness whose testimony he is not certain will aid his side in the case. The experts found in our courts are, therefore, men of pre-ascertained views, brought into court because they are advocates of some particular theory, which is needed to sustain the complaint or defense. Hence it is that in almost all our criminal trials experts are found swearing positively and directly contrary to one another. In the Stokes-Fisk case, for example, experts were equally divided in opinion as to whether the deceased died from the effects of the wound or the manner in which the ball was extracted." And to harmonize these opposing views of experts, and to inform the jury of the scientific principles which they shall recognize in making up their verdict, this duty rests upon judges who, not being themselves medical experts, do not possess the knowledge necessary to determine real medical science from speculative theories. Medical experts should be medico-legal advisors to assist the court in forming a decision upon medical questions. But these advisors are hired in the interest of one party, and paid for their services by the interested party, consequently their testimony is not unbiased, and is always so contradictory that the legal profession looks with contempt upon medical witnesses. Judge Davis, of Maine, is quoted as having said: "If there is any kind of testimony that is not only of no value, but even worse than that, it is, in my opinion, that of medical experts."

Thus, the medical men have to bear the blunt of ridicule and contempt, which truly and justly belongs to the absurd, monstrous system in vogue in our courts. This needs a radical change, and to accomplish that with intelligence and success, we must study, as a model system, that of Germany. "In Germany alone has medical jurisprudence received a proper estimate of its worth. It was introduced into the German universities at their foundation, and made a part of medical education. The expert is made a government officer, holding his position because of his peculiar practice, skill, and fitness." * * * "Their appointment is continuous, and they owe it to neither party, but to the State. They make personal investigation of the facts in every case. Power is given them to take testimony. They may call experts as counsel if they wish, but these experts must have certificates, received from State examinations, of their skill in the specialty whose principles it is desired to apply. The facts and proofs, and the conclusions arrived at by the expert tribunal, are shaped into a written report, which is carefully preserved along with the report of the subsequent proceedings. So far as the court is concerned this report is conclusive." If the parties interested be dissatisfied with the conclusions of this expert tribunal, they may appeal to a second medical commission, and from its decision an appeal may be taken to the Supreme Medical Commission, comprising men of national reputation. The German government "recognizes the fact that experts stand to the court in the relation of assistants. It, therefore, denies to those who are interested the choice of these assistants. The educational requirement renders the expert competent. His appointment by the State ought to render him impartial." The adoption of a similar plan is strongly advocated by Dr. Philips in the remodeling of the present method of precedure.

Dr. J. W. Hamilton gives us a very interesting report of cases of "Maxillary and Naso-pharyngeal Tumors."

Dr. C. S. Muscroft speaks very highly of sub-sulphate of iron as a local remedy for erysipelas, acute and chronic ulcers of the lower extremities, also "for injections in puerperal fever, attended with vitiated discharges from the uterus and vagina;" and, "in ulcerations of mucous linings of the nose, attended with fetid discharges."

Dr. Thad. A. Reamy illustrates by way of clinical reports the use of the curette in certain forms of uterine diseases. His experience with the instrument has been "extensive and eminently satisfactory as to safety and efficiency."

Dr. R. L. Sweney reports a case of an inverted uterus reverted seven months after its inversion.

Dr. C. H. Read has, for the past seven years, tested the efficiency of quinine as a prophylactic in scarlet fever, "but kept a full record only of twenty-nine cases of exposure." "Of these, twenty took quinine as a prophylactic. Of these twenty, nineteen, or ninety-five per cent., escaped. Of the nine who took no quinine, none escaped."

The report on the progress of ophthalmology during the year 1877, by Dr. D. B. Smith, may be recommended as a model to the attention of all committees of medical societies charged with the duty of making an annual report on the progress in the various branches of medical science.

2. It is a pity that the *Pennsylvania State Medical Society* does not appreciate the advantages of solid book-binding. Its transactions deserve a better fate than to be sent out with a simple paper cover and with the leaves stitched so loosely that after a short use they are out. Volumes of 500 large octavo pages have outgrown that embryonic stage of a book, called *brochure*, and, therefore, should not be dressed as a baby but treated as a full-grown adult.

The present volume contains addresses by eminent men, which deserve more than passing attention.

President D. Hayes Agnew selected for his address the very suggestive theme of "Errors of Diagnosis." Relating some famous cases of this kind, he endeavored to show that in the most of these cases errors of diagnosis could have been avoided, and their occurrence was due to superficiality in examination on the part of the surgeon. "Surgeons of large experience, by their great familiarity with disease, are often governed more by their intuitions in deciding upon the nature of a case, than by the tedious and cautious plan of analysis practiced in the earlier years of their career." "Such knowledge tends to engender a spirit of over self-confidence which may lead to fatal errors."

A large number of errors of diagnosis may be placed to the account of ignorance. To this source must be traced instances of undetected luxations of the humerus, cases in which hernia, varicocele, and hydrocele are confounded with one another, and similar blunders.

Errors of diagnosis not unfrequently originate perplexing litigations which might often be obviated if medical men were less officious and more reserved in expressing an opinion upon the skill of a professional brother. Every physician must blush over this statement of Dr. Agnew: "In almost every suit for malpractice with which I am acquainted, singular as it may appear, there has been a doctor at the root of it." This could not be the case if every physician adopted the policy advocated by the essayist. "I do not wish to be understood as advocating a policy which would shield any member of our profession from the just consequences of ignorance, recklessness, or neglect; but do say that any medical man who ventures to assail the professional skill, or to lessen the confidence of the public in a brothor practitioner, without being conversant with all the facts in dispute and having the most conclusive evidence of the correctness of his own diagnosis, deserves, in the highest degree, public censure, and should be ostracized by the entire body of medical men."

Dr. W. Goodell delivered a very interesting lecture on "Laceration of the Cervix Uteri," giving a graphic description of the local and constitutional disturbances this lesion may produce. It is an important factor in the production of uterine disorders and a common cause of sterility. The treatment, medicinal and operative, of fresh and old lacerations is dwelled upon very extensively, and his own mode of performing the operation fully described. Those interested in the subject will read this address with pleasure and profit.

Dr. Samuel W. Gross favored the society with an address in surgery, selecting for a topic, "The Rational Treatment of Stricture of the Urethra." The substance of his lecture is incorporated in the following axioms:

1. The rational treatment of stricture of the urethra consists in restoring the natural expansibility, or caliber, of the affected portion of the canal.

2. Before any operation, having this end in view, is practiced, the sensibility of the urethra should be obtunded, lest it resent the violence to which it is about to be subjected.

3. After the spasm and hyperæsthesia have been relieved, the caliber of that portion of the urethra in which the stricture is located, should be estimated by the urethrometer, with the view of bringing the affected portion to that standard.

4. Internal urethrotomy from behind forwards is the most effectual mode of accomplishing that object.

5. The meatus should not be interfered with, provided its circumference is eight millimeters less than than of the spongy urethra.

Dr. S. D. Risley gave a clinical analysis of one hundred and fifty-three cases of iritis. He found iritis had furnished four per cent. of all the cases of eye diseases coming under his care. Only fifteen cases were seen during the first week of the disease, in a very large number the true nature of the disease had not been recognized.

*Syphilis* stood out prominently as an efficient cause of iritis; *sixty* cases of the 153 were plainly due to this cause, and were for the most part associated with the secondary symptoms of the disease. *Four* cases were undoubtedly rheumatic, and *twelve* traumatic. In the remaining cases no cause was stated in the record. The *serous iritis* occurred in but four cases; all other cases showing the *parenchymatous* or *plastic* form of iritis.

Beside the use of atropine, local depletion and opiates ("Dover's powder in full doses, or morphia hypodermically"), mercury was freely employed. And it is worth recording, the opinion of Dr. R., founded upon the careful study of so large a number of cases: "I have repeatedly, for many days, used strong solutions of atropia in the most persistent and vigorous manner, assisted by the local abstraction of blood, with no effect upon the synechiæ, other than slightly stretching them; but no sooner were the gums spongy and a slight mercurial fetor of the breath, than the adhesions lost their tenacity, either melting down and disappearing as by magic, or becoming friable, lost their hold upon the capsule, and the pupil being free, expanded widely under the action of atropia."

"This beneficial action of mercury in iritis is by no means confined to cases of syphilitic iritis. In any case of acute iritis, with marked tendency to plastic exudation, the surgeon does not do his whole duty who fails to use mercury to the first manifestation of its systemic effects, except, possibly, in the comparatively rare cases where such an acute attack is obviously due to a gouty or rheumatic diathesis."

"The method of administration has many times seemed a matter of importance. Inunctions have certainly, in my experience, proved of greater value than the administration by the mouth in any form."

There are a number of other excellent papers in this volume, which contain many practical suggestions. Dr. William Pepper contributed a "Clinical study of Catarrhal Inflammation of the Bile-ducts, with Remarks on the Use of Nitrate of Silver in its Treatment." Dr. Lawrence Turnbull reported "Three Cases of Disease of the Mastoid Process," to show the difference in the clinical pictures of: (*a*), Inflammation of the external periosteum; (*b*), Acute inflammation of the mucous membrane, followed by accumulation of pus in the mastoid cells; (*c*), Chronic sub-acute inflammation of the mucous membrane, with sclerosis. The same author read a paper on his "Experiments and Observations on the Physiological Properties of Hydrobromic Ether."

Dr. E. Dyer contributed two short papers "On Sympathetic Ophthalmia," and "On the Metric System in Medicine."

"Anæsthesia in Parturition," by A. H. Halberstadt; "Rosacea," by John V. Shoemaker; "Psoas Abscess Simulating Nervous Affections," by Chas. K. Mills; "Some Remarks upon the Descent of the Testes, and the Formationof Oblique inguinal Hernia and Hydrocele," by O. H. Allis, conclude the series of original communications; they, with reports of committees, fill the first two hundred and fifty pages of the volume; the second half is given to the reports of the county medical societies. F. C. H.

Article XVIII.—Deterioration and Race Education. By Samuel Royce. Boston: Lee & Shepard, Publishers, 1878.

More than once, since opening the covers of this volume, we have been disposed to return it, as irrelevant to the subjects which come legitimately within the scope of a medical reviewer. There are, however, certain points of contact between the book and a class of subjects with which the physician is continually made familiar, which will justify a criticism of a portion, at least, of its contents.

The work appears to be the outcome of the researches and speculations of a professional philanthropist. Like the majority of kind hearted people, blessed with a scanty knowledge of men and things, emerging for the first time from the happy seclusion in which they have been cradled, he looks about him in the whirling throng of human creatures, and remarks with sympathetic pain the wretchedness which overwhelms so many of his fellow beings. The farther he looks, the more suffering he beholds. It certainly was not so during his childhood in the little nook among the hills where he was born; consequently the race has fallen upon evil days, and the times must be growing worse. He begins to read, and his discoveries appal him. Everywhere he finds for each year a higher figure for every form of crime, disease and death. In every State of the Union new prisons and new asylums are springing out of the ground, and yet the number of unhoused lunatics and paupers is continually growing. It must be so! Statistics prove it! Criminals are more numerous! Hospitals are more and more crowded! The insane are increasing! Poverty is rising like a flood! There are the figures, and they can not lie! Such is the course of reasoning by which this author demonstrates the deterioration of the race. He has raked together an immense amount of statistical information of this kind; but so thoroughly is it dissociated from qualifying facts, that it is utterly useless and misleading in its character, for he makes no reference to the fact that the population has greatly increased between the years under comparison, and that improved methods of collecting statistics have swelled all later returns out of due proportion with the earlier tables. It is an error precisely similar to the blunder which one would commit were he to contrast the number of insane persons in the Cook County Asylum, fifteen years ago, with the number now cared for in that institution. Fifteen years ago there were not more than a dozen lunatics in the almshouse at Jefferson. The population from which they came was about one hundred and fifty thousand souls. That population has now increased four-fold, but the lunatics in that almshouse to-day, since the erection of the new building, number more than four hundred persons. In view of this fact, if we allowed ourselves to employ the logic of this book, we might justly infer that Cook county will soon be inhabited by insane people alone.

The fact is, that statistics are not to be trusted, for the information which will enable any one to decide whether the race as a whole is deteriorating. It is easy enough to trace the course of a family, or a tribe. The stalwart pioneers who have expended all their energies in grubbing stumps and fighting Indians in a malarious climate, are very likely to be succeeded by a puny offspring. Hardy laborers, transplanted from the hay field into the steaming atmosphere of a cotton mill, usually die young, and are followed by a scrofulous line of descendants. Ancestral piety avails little as a protection against the seductions of newly gotten wealth. But these are accidental and individual phenomena. If we would learn the truth of the matter, we must study the history of the world, and must compare the centuries with each other. Such investigation gives results very different from the conclusions of our author. Perhaps this may be one reason why he manifests such a contempt for those classical studies which make us acquainted with the ages that have passed, and are unknown to the majority of mankind.

This error of hasty generalization is the fault which vitiates all the conclusions of this book. It contains a vast heap of unverified assertions, with very little regard for logical sequence. The race, says the author, is growing continually worse, as a consequence of wrong methods of education and occupation for the masses. Our schools are devoted to training the young in a body of useless knowledge, such as history, reading, mathematics, languages, music, etc.; consequently, when young people leave school they are unfitted to earn their own living, and are never willing to do any useful work, preferring rather to "live by their wits." Our present school system should therefore be destroyed, and in its place should be provided kindergarten schools for small children, and manual labor schools for those of larger growth, in which young people should be taught the trades and the principles of agriculture. When the pupils have graduated from these places of learning, they will be so well skilled in all mechanical and agricultural arts, that of course they will always find abundant occupation and high wages. But since many of the trades are notoriously unhealthy, capitalists must be compelled to make them healthy. And since it has never been found possible to house the working people in factory towns without a great amount of over-crowding and misery, these operatives must all be furnished with houses in the country, where they can all have their gardens, and their pigs and poultry. But since the railway service necessary to carry laborers from the city factories to their rural homes is expensive, the railway owners must be compelled by law to carry such passengers for a nominal sum, or else the factories must all be moved out of town into the healthful country air. Inasmuch as these great improvements will not be undertaken by ordinary "bloated bond holders," laws must be made, compelling them to conduct their business on philanthropic principles, and we must have an army of inspectors and other officials, to carry out these beneficent laws.

Such, in short, is the argument of this book. It is the same line of reasoning with which one becomes so familiar in the volumes which record the proceedings of our Social Science Congress, Public Health Associations, Board of Health meetings, and Philanthropical gatherings generally. It is the argument which is exceedingly popular with all benevolent people who feel acutely over sufferings which they witness, but who have not the knowledge with which to discover the true causes of such misery, nor the patience and energy requisite to undertake the measures necessary for their effectual relief. Something short, sharp and decisive is what they desire. *Stamping out* is their favorite phrase, and they care little how much evil may be done, if they are persuaded that good will come of it.

In all this, however, the benevolence which animates these people is beyond praise. The thing to regret is the misdirection of their energies. The improvement of the condition of the human species is their object, and it is the most laudable object in the world. But they mistake the way to the accomplishment of their desires, when they think to ameliorate the condition of mankind through the strong arm of civil government. The effort to secure such a result by the interposition of civic power has been tried again and again, and it has always failed. The real progress and improvement of the race has always been effected by other agencies, which have usually been compelled to array themselves in active opposition to existing governments. The entire history of the growth of liberty—which is the greatest blessing a human being can enjoy—is the record of a continual warfare against constituted governments. We in this country have secured the largest amount of liberty ever possessed for any length of time by any people, but we are by no means thoroughly emancipated yet. There is a constant effort on the part of the office-holding and office-seeking classes to increase their own profits and power at the expense of citizens. There is a constant tendency on the part of these classes to extend the functions of government to affairs with which it has no rightful relation in a free society. Such being the fact, it is a reason for regret when we discover the philanthropical portion of the community unwittingly seeking to aid the official classes in their insidious attacks upon the liberty of society.

Did time and space permit, it would be profitable to show the evils which always result from governmental interference with affairs outside of its legitimate province. Good government is that which busies itself solely with the protection of the rights of its subjects—which secures for each individual the largest amount of liberty consistent with the liberty of his fellows. Just as soon as government undertakes other functions, its weight begins to bear oppressively upon individuals and classes in society. As soon as it becomes apparent that the strong arm of the State can be secured for the advantage or disadvantage of favored or disfavored individuals, a struggle begins to obtain in the direction of that power for private ends. The highest success in this effort produces a despotism—a monster from which we all recoil—but the community is full of people who are anxious to get acts of despotic departure from good government performed for their individual welfare. Every such act is not only an injury to the community and to the individual who fancies himself thus benefited, but it also works out its own final failure to accomplish the end desired. To make this clear, let us review a few out of the innumerable examples which might be adduced in proof of this proposition. English merchants during the last century thought it would be greatly to their advantage to have the trade and manufactures of America regulated by act of Parliament for their own benefit. The result was a bloody war, an addition of one hundred million pounds to the national debt of England, and the loss of the finest colonies ever possessed by the crown. Governmental interference with the natural laws of commerce was not beneficial in that case to the traders who were so clamorous for law in their own behalf.

Scarlet fever is a dangerous disease, of a rather communicable character. Not long ago there was a tremendous outcry for governmental interference to *stamp out* the disease. The result of a great deal of activity on the part of the official guardians of the public health, was, according to the published statistics, to raise the mortality of scarlet fever, in this city, from about ten per cent. to nearly thirty per cent. of the cases. This was the ostensible result. The real result was to produce an extensive concealment of the disease, for fear of the obnoxious and useless measures which are employed in the name of the law of the land. Now, when one reflects upon the fact that a case of concealed disease of this character is more dangerous to a community than a case which is openly confessed, it is easy to see how much benefit we derive from such official interference. Syphilis is another terrible scourge of the human race. There are plenty of people —doctors too—who are crying out for a governmental inspection and purification of prostitutes, in order to restrict the prevalence of venereal diseases. Such people are very fond of quoting the results of such measures in the army and the navy, and have much to say about the vast diminution of suffering among the poor girls who are subjected to the periodical visits of the governmental inspectors. They are not in the least chary of their sneers at the "uncognid," as they call those who presume to question the wisdom of the measures which they propose, and are evidently quite unconscious of the fact that they are all the time writing themselves down in the catalogue of *ignorant sentimentalists: ignorant* because unacquainted with the natural laws which control such things, and *sentimentalists* because they allow their sympathy with the suffering of a certain worthless minority to close their eyes to the highest interests of the vast majority of mankind. This is not the place for a full discussion of the regulation of prostitution. It is sufficient for us to remind the reader that it has been again and again proved by the highest authority in such questions, that the hopes of prevention of syphilis by official regulation are fallacious; and that, even if the results of military experience in barracks, where soldiers are made to eat, drink, and copulate to the sound of drum and fife under the ever present eye of an officer, could be secured in the private life of every citizen, the consequent debasement of public morals would be sufficient to condemn the whole thing.

Again, if we consider the results of the administration of charity by the Government, we find that the intrusion of the civil power has been only disastrous in this field of effort. Consider the waste and the corruption which characterizes every branch of public charity administered by the government. Consider the complaints of the tax-payers who give to the poor through the official channels because they *must*, and not because they desire the relief of misery. Consider, also, the wide separation between rich and poor, which is thus intensified, for when the wealthy merchant has paid his tax for pauper relief, he will not listen to the story of distress which is related at his door. We shut up our hearts and our purses when our taxes have been paid; consequently, we lose all the humanizing influences which flow from the judicious exercise of individual charity. Consider our public asylums and hospitals—how much more they cost than similar institutions supported by private benefaction—how difficult it is to secure in them the services of *good men*, while a private charity can secure in its management and service the best talent of the country. It is not too much to assert that our whole system of public charity, based upon governmental supervision, is productive of as much injury as good. It is the outcome of the impatience of people, who cannot wait for the slow operation of natural law, but must force an unhealthy and precocious growth by the aid of government. That the results of such methods should be fraught with evil is only what might be expected when we consider the source of governmental power. All civic governments reflect the masses of the population over which they are placed. The intellectual and moral level of the masses in even the most enlightened nation is very low, consequently the government which represents such masses cannot exhibit any very exalted tendences. It does not, therefore, furnish the right kind of motive power for the advancement of projects which look to the elevation and improvement of the race. Nature has provided a sufficient motive power in the moral and intellectual sense of the race; and it is to this that philanthropists must be content to appeal, if they desire a real and lasting success.

We have numerous societies for the benefit of people. One more organization is imperatively required—a society for the enlightenment and restraint of philanthropy. H. M. L.

